# The Role of Pathological Aging in Cardiac and Pulmonary Fibrosis

**DOI:** 10.14336/AD.2018.0601

**Published:** 2019-04-01

**Authors:** Lucy A. Murtha, Matthew Morten, Michael J. Schuliga, Nishani S. Mabotuwana, Sean A. Hardy, David W. Waters, Janette K. Burgess, Doan TM. Ngo, Aaron L. Sverdlov, Darryl A. Knight, Andrew J. Boyle

**Affiliations:** ^1^School of Medicine and Public Health, The University of Newcastle, Callaghan, NSW, Australia.; ^2^School of Biomedical Sciences and Pharmacy, The University of Newcastle, Callaghan, NSW, Australia.; ^3^Hunter Medical Research Institute, New Lambton Heights, NSW, Australia.; ^4^University of Groningen, University Medical Center Groningen, Department of Pathology & Medical Biology, GRIAC (Groningen Research Institute for Asthma and COPD), Groningen and W. J. Kolff Research Institute, The Netherlands.; ^5^Respiratory Cellular and Molecular Biology Group, Woolcock Institute of Medical Research, Glebe, NSW 2037, Australia.; ^6^Discipline of Pharmacology, The University of Sydney, NSW 2006, Australia.; ^7^Department of Anesthesiology, Pharmacology and Therapeutics, University of British Columbia, Canada.; ^8^Adjunct Professor, Department of Medicine, University of Western Australia, Australia.; ^9^Research and Innovation Conjoint, Hunter New England Health District, Australia

**Keywords:** Cardiac fibrosis, pulmonary fibrosis, mitochondrial dysfunction, senescence, autophagy, inflammaging, heart, lung, aging

## Abstract

Aging promotes a range of degenerative pathologies characterized by progressive losses of tissue and/or cellular function. Fibrosis is the hardening, overgrowth and scarring of various tissues characterized by the accumulation of extracellular matrix components. Aging is an important predisposing factor common for fibrotic heart and respiratory disease. Age-related processes such as senescence, inflammaging, autophagy and mitochondrial dysfunction are interconnected biological processes that diminish the regenerative capacity of the aged heart and lung and have been shown to play a crucial role in cardiac fibrosis and idiopathic pulmonary fibrosis. This review focuses on these four processes of aging in relation to their role in fibrosis. It has long been established that the heart and lung are linked both functionally and anatomically when it comes to health and disease, with an ever-expanding aging population, the incidence of fibrotic disease and therefore the number of fibrosis-related deaths will continue to rise. There are currently no feasible therapies to treat the effects of chronic fibrosis therefore highlighting the importance of exploring the processes of aging and its role in inducing and exacerbating fibrosis of each organ. The focus of this review may help to highlight potential avenues of therapeutic exploration

Fibrosis is the formation of fibrous connective tissue in response to injury. It is characterized by the accumulation of extracellular matrix (ECM) components, particularly collagen, at the site of injury. Fibrosis is a vital component of wound healing and tissue repair, although, continued activation is highly detrimental and a common pathological process in cardiovascular and respiratory disease [1]. The primary cellular and molecular actions of fibrotic diseases share many functional similarities, despite differences in etiology and clinical outcome [2]. At its core, chronic fibrosis is defined by the overgrowth, hardening and scarring of tissues due to continuous wound-healing which can effect multiple organ systems including but not limited to the heart, lung, kidney, liver, and skin [3]. We previously reported that fibrosis of the heart and lung make up a significant proportion of fibrosis-related deaths [1], in this review we will focus on the role of aging in the heart and lung.

Aging is a predisposing factor for cardiac and pulmonary fibrosis, with the prevalence of heart failure and fibrotic respiratory diseases such as idiopathic pulmonary fibrosis (IPF) increasing dramatically with advancing age [4, 5]. The aging of cardiac and lung tissue ultimately results in structural remodeling of the extracellular matrix caused by alterations in the concentration and organization of ECM components such as collagen and elastin [5-7]. Biological aging is accelerated by the cumulative damage and stress that occurs during a lifetime. This premature aging is particularly pertinent to the pulmonary system, which is subjected to lifelong challenges by airborne pollutants, particulates and pathogens [8]. Similarly, due to the high metabolic demand of the heart, large mitochondrial population and infrequent cardiomyocyte turnover, the heart is also highly susceptible to cumulative oxidative damage and stress with age [9]. Cellular and immunological changes occur concomitantly with age-related tissue remodeling.

There are a great many hallmarks that represent common denominators of aging, such as stem cell exhaustion, genomic instability, telomere attrition, epigenetic alteration and loss of proteostasis [10]; in this review we focus on four processes of aging which play an integral role in fibrosis. Senescence, inflammaging, compromised autophagy and mitochondrial dysfunction are interrelated processes, which reduce the regenerative capacity of the aged heart and lung, and have been shown to be involved in cardiac fibrosis and IPF [5, 11, 12]. As a consequence, challenges to an aging heart or lung are more likely to lead to pathological tissue remodeling rather than wound resolution and tissue restitution. This is exemplified in experimental models that show cardiac fibrosis in mice post-myocardial infarction increases with age [13]. Similarly, pulmonary fibrosis in experimental lung injury is exacerbated by aging [14].

**Figure 1.**
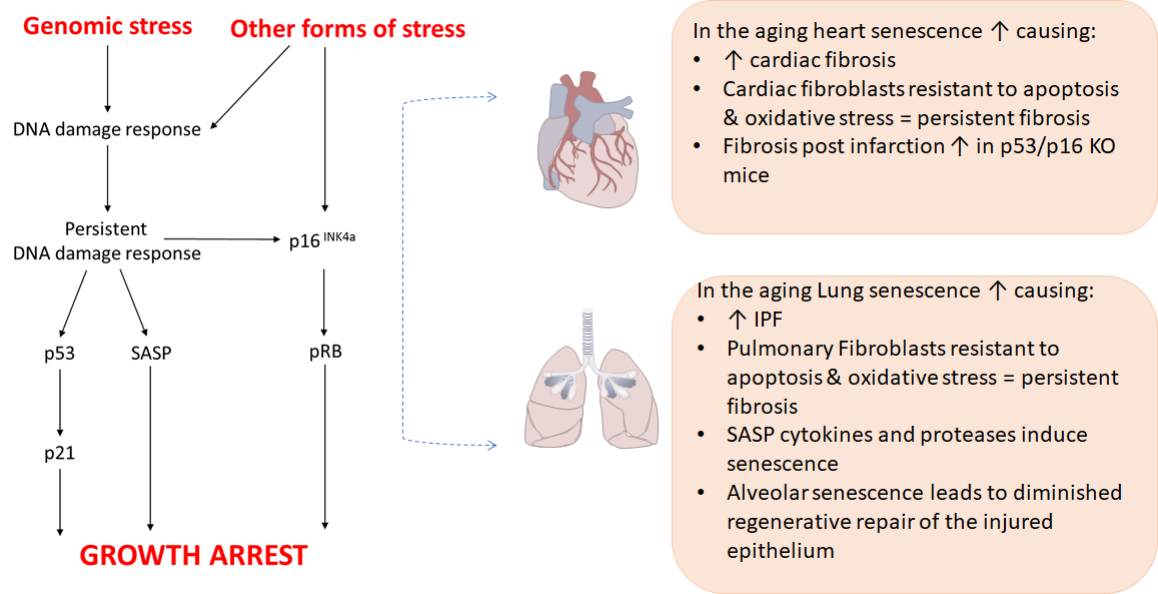
Regulation of senescence growth arrest and the senescence-associated secretory phenotype (SASP) in the aging heart and lung Stresses inducing senescence vary depending on the context, resulting in a variety of effector pathways. However, there is considerable overlap in processing of the stress-response signal and activating effectors of senescence, with a common final outcome, arrest of cell growth.

## 1.Cellular senescence

Cellular senescence is an important hallmark of aging [8]. Senescent cells are refractory to mitogenic stimuli, a consequence of increased levels of the cell-cycle arrest proteins p16^INK4a^ and p21^CIPI^ [15]. These cells also acquire a senescence associated-secretory phenotype (SASP), an important pathological feature of senescence, characterized by the increased production of inflammatory and fibrogenic mediators [16, 17]. Cellular senescence is a result of the induction of the p53-p21 and/or p16-pRB pathways following a DNA damage response (DDR) (Fig. 1). One form of senescence, termed ‘replicative’ senescence, is a consequence of chronological aging involving telomere shortening. Telomeres are comprised of repetitive DNA sequences at the end of chromosomes which protect DNA from damage. In somatic cells, telomeres shorten with each successive cell division until DNA damage and senescence eventuate. ‘Stress-induced’ senescence, also involving a DDR, is evoked by cellular insults such as oxidative stress, radiation, mutagens and SASP cytokines [15-18] as described in Figure 1. Intracellular abnormalities such as defects in the telomerase complex also contribute to senescence. The contribution of senescence in cardiac and pulmonary fibrosis is evidenced in clinical and experimental studies. Markers of senescence are detected in resident cells of human fibrotic heart and lung *in situ* and after isolation *in vitro* [18-20]. Senescence is evident in cardiac tissue of aged mice at baseline and increases in young and aged mice following myocardial ischemia-reperfusion injury [20-22]. Cellular senescence is also higher in lung tissue of older mice than young mice at baseline, and increases in experimental lung injury and fibrosis [23]. The selective clearance of senescent cells in transgenic mice attenuates age-related deterioration of cardiac and lung tissue [24, 25]. Furthermore, accelerated senescence prone mice are more susceptible to experimental cardiac or pulmonary fibrosis than accelerated senescence resistant mice [26, 27]. Additionally, senolytic agents, a new class of small molecules which target senescent cells, and the selective ablation of senescent cells have been demonstrated in transgenic ‘Ink Attac’ mice to attenuate lung fibrosis following bleomycin instillation [28]. Indeed, several senolytic agents, including sirolimus, acarbose, and nordihydroguaiaretic acid (NDGA) have been shown to extend the lifespan of mice by the National Institute on Aging Interventions Testing Program.

The roles of cellular senescence in cardiac and pulmonary fibrosis are complicated, particularly as cellular senescence is protective in certain situations (e.g. when cells become cancerous and during the early stages of tissue injury). Contributing to the ambiguity, cardiac fibrosis post-infarction has been shown to be greater in p53 and/or p16 knock out mice than wild type mice [29, 30]. However, these studies were conducted with young mice. In experimental models of cardiac or pulmonary fibrosis using aged mice, the targeting of senescence markers or mediators including Nox4, MMP-9 or plasminogen activator inhibitor-1 was protective [14, 20, 31]. In explaining the increased susceptibility of aged mice to pulmonary fibrosis, Thannickal and colleagues proposed that pulmonary fibroblasts in young mice don’t undergo complete senescence following injury, allowing for apoptosis and fibrosis resolution [20]. However, in aged mice, the fibroblasts become completely senescent after injury, leading to apoptosis resistance and a persistent fibrosis. In support, fibroblasts derived from aged cardiac or lung tissue are more resistant to apoptosis and oxidative-stress induced toxicity than fibroblasts of younger tissue [14, 18, 20, 32]. The acquisition of the SASP is another important feature of senescent fibroblasts that contributes to fibrosis in disease. The hypersecretion of proteases, cytokines and fibrogenic mediators by senescent fibroblasts would have a large impact on nearby non-senescent resident cells, potentially perpetuating the fibrotic response. In cardiac disease, fibroblast-derived mediators such as TGF-β induce cardiac myocyte hypertrophy, which leads to apoptosis via induction of apoptosis signaling kinase-1 [33]. In IPF, SASP cytokines (e.g. IL-6) and proteases (e.g. urokinase plasminogen activator) potentially induce fibroblast and epithelial cell senescence in nearby undamaged tissue, or re-inforce the senescent phenotype in an autocrine manner [16, 34].

Overall, cellular senescence can be considered a beneficial compensatory response to the damage caused by fibrosis and aging when tissues of the heart and lung exhaust their regenerative capacity. However, the ambiguity regarding the roles of cellular senescence in cardiac and pulmonary fibrosis emphasizes the demand for further research to fully elucidate the potential therapeutic implications behind modulation of senescent pathways. Although, with the advent of senolytics that either suppress senescence or selectively kill senescent cells, the goal of delaying, preventing and alleviating age-related diseases suggest that it may be possible in the future.

## 2.Inflammaging and immunosenescence

Inflammaging and immunosenescence are interconnected age-related processes that also have important roles in cardiac and pulmonary fibrosis. Inflammaging describes elevated baseline levels of inflammatory markers in tissues and the circulation of the elderly in the absence of an immunologic threat. Sustained increases in the levels of inflammatory cytokines (e.g., IL-6 and TNF-α), differentiated lymphocytes and autoantibodies in heart and lung tissue, as well as the circulation, mark inflammaging in aged humans and rodents [35-38]. As a result of prolonged antigenic stress, inflammaging contributes to immunosenescence, a delayed but protracted immune response to challenge in the elderly. In immunosenescence, innate and adaptive immune responses such as the phagocytic clearance of cellular debris and lymphocyte activation respectively are diminished. Immunosenescence and a corresponding loss in immunoregulation is implicated in both fibrotic cardiovascular disease (CVD) and IPF [37, 39]. Aberration of toll like receptor signaling, which is important in the recognition and initiation of adaptive immune responses, occurs in CVD and ‘rapidly progressive’ IPF [40, 41]. A compromised adaptive immune system in biological aging is particularly pertinent in myocardial infarction, as the impaired clearance of dead myocytes leads to maladaptive tissue repair and subsequent pathological tissue remodeling [38]. Changes in T-helper cell populations including the down-regulation of CD28 (a co-stimulatory molecule for T cells) on CD4 cells also occur in CVD and IPF [42, 43]. Increases in circulating autoantibodies and immunocomplexes are another aspect of immuosenescence associated with CVD and the onset of IPF [44, 45].

**Figure 2.**
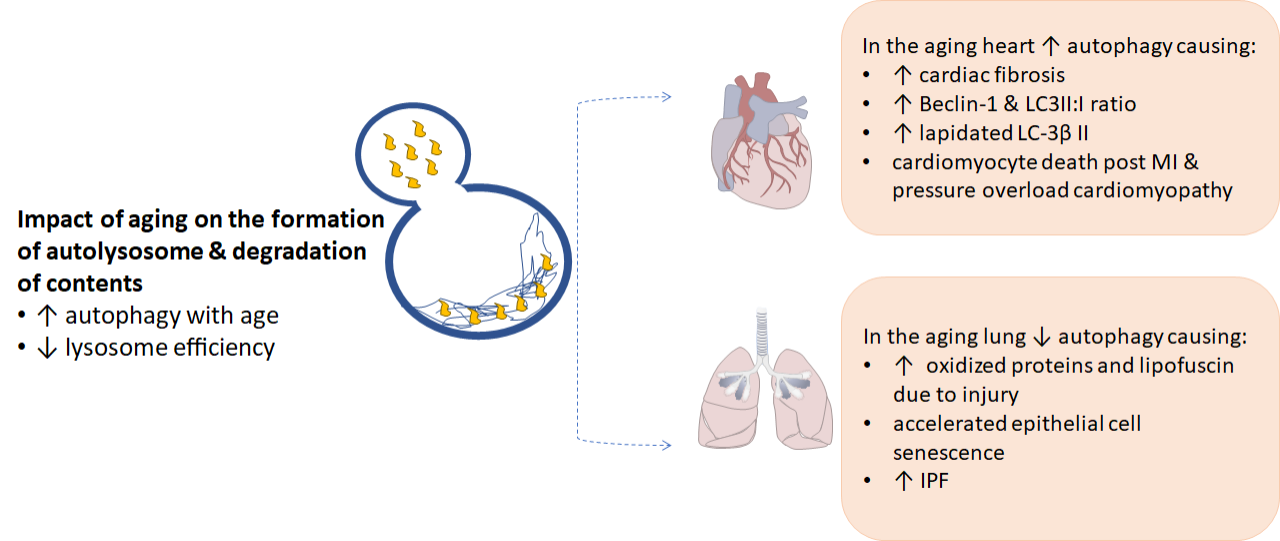
Impact of aging on the formation of autolysosome & degradation of contents Aging increases the cardiomyocyte’s need for autophagy to maintain intracellular homeostasis, but simultaneously reduces the activity of lysosomes and thereby inhibits autophagic flux. The effects of aging on autophagy are opposing in the heat and the lung leading to variable pathological outcomes.

## 3.Autophagy

Autophagy is an intracellular degradation process whereby components of the cytoplasm are degraded in lysosomes. Autophagy is required for physiological homeostasis and is largely considered a protective pathway. Several forms of autophagy have been described in mammalian cells, macroautophagy, microautophagy and chaperone-mediated autophagy. The term autophagic flux refers to the whole process of autophagy, including autophagosome formation, maturation, fusion with lysosomes, subsequent breakdown and the release of macromolecules back into the cytosol [46]. It is often described as a measure of autophagic degradation activity and a number of methods are currently utilized to assess autophagic flux [47]. Macroautophagy is the primary pathway and involves the turn-over of redundant or damaged cells and proteins, facilitated by autophagosomes. This process is tightly controlled by autophagy-related proteins (e.g. Atg5, Atg7, microtubule-associated protein 1 light chain 3 (LC3), Beclin-1) [48]. One of the key regulators of macroautophagy in mammalian cells is mTOR (mammalian target of rapamycin) kinase [49]. Inactivation of mTOR initiates autophagy. Macroautophagy is considered the first line of attack for cellular stress, with chaperone-mediated autophagy increasing after 6-8 hours of prolonged stress [50] (Fig. 2).

Diminished autophagy has been associated with several age-related diseases including cancer, neurodegeneration, metabolic defects and IPF [51, 52]. The specific mechanisms associated with this age-related autophagy decline remains unclear. Research suggests that a reduction in autophagy accompanies the age-related deterioration in cardiac function following myocardial infarction [53] and accumulation of dysfunctional mitochondria [54, 55], as discussed below.

Several studies have demonstrated that autophagy activity is reduced in IPF lungs and suggest that this decrease may induce pro-fibrotic response [56-59]. Patel *et al* demonstrated that autophagy was not induced in experimental pulmonary fibrosis despite activation of pathways known to promote autophagy [57]. They suggested that TGF-β mediated autophagy impairment may represent a mechanism for the promotion of fibrogenesis in IPF. Romero et el found that aging contributed to lower autophagy induction in primary IPF lung fibroblasts compared to young and aged-matched controls, and that this was activated by the mTOR pathway [58]. Similarly, Sosulski *et al* demonstrated in animal models of pulmonary fibrosis that older mice were characterized by reduced autophagy in response to lung injury, that autophagy diminished with corresponding elevated levels of oxidized proteins and lipofuscin in response to lung injury in old mice, and they suggest reduced autophagy may contribute to the promotion and/or perpetuation of pulmonary fibrosis [59]. Furthermore, Araya *et al* demonstrated in human IPF lung fibroblasts that autophagy inhibition can induce accelerated epithelial cell senescence and myofibroblast differentiation [56].

In stark contrast to the lung and other fibrotic organs, it appears that an increase in autophagy is a major regulator of fibrosis in the age-related diseases of the heart. Autophagy has been described as a critical factor in cardiac fibrosis [60-62] however the specific mechanisms by which this occurs are currently under-represented in the literature. Boyle *et al* demonstrated that the cardiomyocytes of aging mice displaying cardiac dysfunction, fibrosis and hypertrophy exhibited an increase in autophagic vacuoles, higher Beclin-1 expression and increased LC3II:I ratio compared to young healthy mice (18 vs 2 months) [63]. Further, Gupta *et al* demonstrated that significant upregulation of autophagy, as indicated by increased expression of lipidated LC-3β II, occurred concomitantly with fibroblast to myofibroblast conversion [61]. They also demonstrated that pharmacologic autophagic inhibition repressed the myofibroblast conversion. In support, Hariharan *et al* demonstrated that cardiomyocyte death and pathological remodeling following experimental myocardial ischemia/reperfusion injury and pressure overload cardiomyopathy was associated with significantly increased autophagy [62]. They also demonstrated that inhibition of the pro-autophagic Beclin-1 alleviated the effects of the remodeling. This is also indicated in humans with Ghavami *et al* demonstrating that human atrial fibroblasts treated with pro-fibrotic stimuli, TGF-β1, had increased autophagy in addition to an enhanced fibrogenic response [60], and Garcia *et al* depicting an accumulation of autophagic vesicles and lipofuscin deposits with electron micrography in the biopsies of the patients undergoing coronary artery bypass surgery [64].

There are multiple lines of evidence suggesting that aging is accompanied by impaired autophagy, and that this process can provoke fibrosis of the heart and lung in both animal models and in vitro models. Therefore, the potential benefit up upregulating autophagy as a major regulator of age-related diseases, specifically in fibrosis, offers a unique treatment option to explore in the future.

## 4.Mitochondrial dysfunction

Mitochondria are dynamic intracellular organelles found in most mammalian cells and are the main source of metabolic energy for these cells. As such, they are important for the regulation of cellular homeostasis, and physiological maintenance of apoptotic signalling pathways [65, 66]. Normal functioning and quality control of mitochondria depends on the careful balancing of two highly regulated and opposing processes: fusion and fission. Fusion results in the formation of a single mitochondrion from independent structures, whereas fission refers to the separation of a mitochondrion into two or more daughter organelles [67]. These processes depend on the separating of mitochondrial proteins and mitochondrial DNA (mtDNA) to enable physiological functioning of the individual organelles [67]. Mitochondria are highly susceptible to the effects of aging, and age-related mitochondrial dysfunction is characterized by alterations in structural and functional properties including mitochondrial enlargement, loss of cristae and the destruction of inner membranes which result in disruption to energy production and mitochondrial signaling [65, 66, 68]. The mitochondrial theory of aging proposes that intracellular reactive oxygen species (ROS), primarily produced by mitochondria as a by-product of cellular respiration, mutates mtDNA. ROS production is known to increase with age, and subsequent somatic accumulation of these mutations are thought to contribute to the aging phenotype [65, 66, 69]. Indeed, mitochondrial-targeted catalase has been shown to reduce obesogenic-stress induced left ventricular hypertrophy [70] and halt age-related ventricular fibrosis, mitochondrial protein oxidation, mDNA mutations [70].

Certain animal models of mitochondrial aging utilize a strain of mice possessing a knock-in of the mtDNA polymerase: polymerase gamma (Polg^D257A/D257A^). The resulting missense mutation causes a decline in 3’-5’ exonuclease activity, which reduces the proofreading efficiency of DNA synthesis, and consequently compromises genetic stability [69, 71]. These homozygous mice accumulate more somatic mtDNA mutations over the span of their lifetime. This corresponds with a significantly reduced lifespan and a progeroid phenotype including typical signs of physiological human aging such as enlargement of the left ventricle and development of cardiomyopathy, alopecia, anaemia, frailty, kyphosis, osteoporosis, sarcopenia, testicular atrophy, weight loss, reduced sub-cutaneous fat, reduced fertility, and a decline in haematopoietic stem cell production [65, 69, 72, 73]. Data obtained from these animals advocates a causative role for mtDNA mutations in physiological aging and indicate this may also contribute to the pathogenesis of fibrotic diseases that develop with age, including various cardiomyopathies, and pulmonary fibrosis.

**Figure 3.**
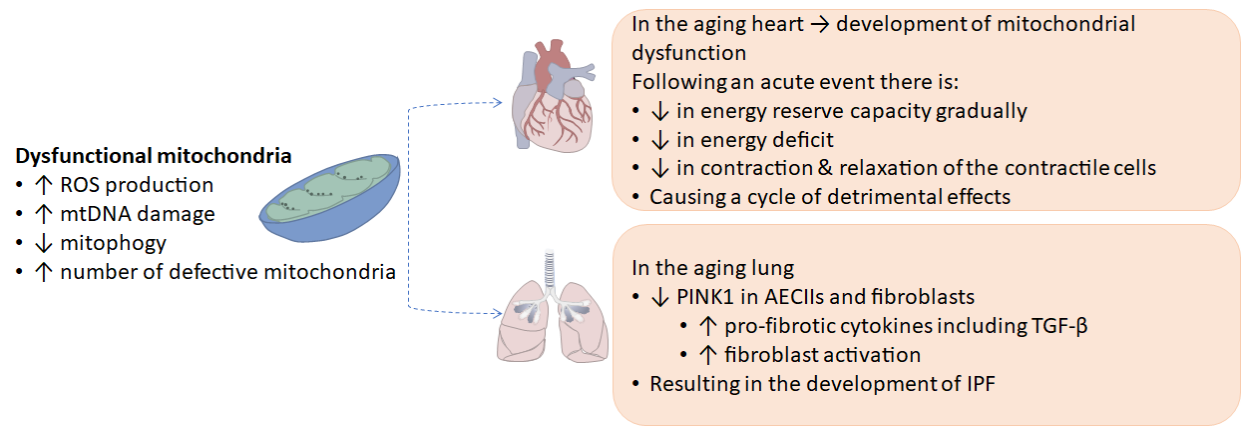
Alterations in dysfunctional mitochondria in the aging or diseased heart and lung Reduction in the ability of dysfunctional mitochondria in the heart and lung to create energy causes an energy deficit that disrupts physiological cellular functioning. Mitochondrial dysfunction in the aging heart and may result in the development of mitochondrial cardiomyopathy or IPF respectively. Mitochondrial dysfunction induced by an acute event in the heart significantly disrupts the contraction and relaxation of contractile cells in the heart and perpetuates a cycle of detrimental effect.

The heart and lung both have a high demand for metabolic activity, and as such are densely populated by mitochondria. Physiological functioning of these organs relies heavily on the regulation of cellular-energetic homeostasis and mitochondrial function [66]. Impaired mitochondrial function has been identified in the heart and lungs of healthy aging individuals and diseased aging patients (Fig. 3) [74, 75]. For example, a reduction in the energy output of cardiac mitochondria, increased ROS production, and suppressed mitophagy has been shown to accompany cardiac senescence induced by physiological aging. This results in the accumulation of defective mitochondria, and interruptions to the processes of fission and fusion [66]. These disruptions can result in the development of mitochondrial cardiomyopathy, a myocardial disorder characterized by abnormal myocardial structure or function in the absence of concomitant coronary artery disease, hypertension, valvular disease, and congenital heart disease [75]. An acute cardiac event on the other hand initiates a sequence of events that results in the gradual decline of the energy reserve capacity of the heart [76]. Several compensatory mechanisms are activated to promote normal functioning and blood circulation; however, when the energy reserve capacity reaches critical levels, these mechanisms are no longer sufficient to maintain physiological functioning and ultimately results in heart failure [76, 77]. This reduces the ability of mitochondria to produce and transfer energy within cardiac cells, thus creating an energy deficit, which leads to a decline in the contraction and relaxation capacity of contractile cells within the heart, and consequently perpetuates a cycle of detrimental effects [76].

Comparably, mitochondrial dysfunction in the form of mitochondrial enlargement, increase of mitochondrial area in aging type II alveolar epithelial cells (AECIIs), and a bias towards fusion has been identified in the aging and IPF lung [68, 74]. There is a general consensus that endoplasmic reticulum (ER) stress induced abnormalities in AECIIs causes irregular re-epithelialisation, secretion of pro-fibrotic cytokines, and fibroblast activation; ultimately leading to the development of IPF [74]. AECIIs are a highly metabolic cell population, which account for approximately half of the total mitochondrial content of the lung [74]. Bueno *et al* demonstrated that AECIIs in highly fibrotic regions of IPF lungs accumulate dysfunctional mitochondria and exhibit impaired autophagy relative to AECIIs from regions of moderate to mild fibrosis, and control donors. This study also established that age (18-24 months) and ER stress alters mitochondrial function in AECIIs isolated from C57BL/6 mice, as determined by reduction in mitochondrial respiration, loss of cell viability and activation of pro-fibrotic responses relative to young (3 months) mice [74]. It has been shown that dysfunctional mitochondria in AECIIs of aging or ER stress exposed animals correlates with the reduced expression of PTEN-induced putative kinase 1 (PINK1), a regulator of mitochondrial homeostasis [68, 74]. PINK1 deficient mice demonstrated apoptosis and TGF-β mediated fibrosis of the lungs, accompanied by structural and functional alterations of mitochondria in AECIIs; consequently, these animals were more prone to lung injury and fibrosis [68, 74, 78]. Asides from AECII cells, Trujillo and colleagues recently showed fibroblasts of lung tissue from IPF patients have increased mitochondrial mass, and that IPF-derived lung fibroblasts, when compared to cells from control donors, release increased amounts of mtDNA in vitro [79]. Importantly, they showed higher levels of mtDNA in the bronchoalveolar lavage and serum of IPF patients, compared to control donors, and serum levels of mtDNA were predictive of all-cause mortality. By virtue of its bacterial ancestry and many specific characteristics such as hypo-methylation, unique structural features and exaggerated susceptibility to oxidative damage, mtDNA can be a potent damage associated molecular pathogen (DAMP) capable of triggering a paracrine inflammatory response by activating inflammasomes (in particular NLRP3) or toll-like receptors (i.e. TLR9) and other DNA binding receptors (i.e. cyclic GMP-AMP synthase [cGAS]) of the innate immune system [80].

Sirtuin 3 (SIRT3) is a mitochondrial protein deacetylase regulator of antioxidant response and mitochondrial homeostasis, known to be an age-related pathogenic mechanism in heart and lung fibrosis. Sosulski *et al* demonstrated reduced SIRT3 expression in the lungs of old mice compared to young mice, as well as in two murine models of pulmonary fibrosis [81]. Mice lacking sirtuin 3 (*SIRT3*KO), have increased acetylation and inhibition of many mitochondrial enzymes and complexes, suppressing mitochondrial function. *Sirt3*KO mice develop spontaneous pulmonary arterial hypertension (PAH) [82] and cardiac fibrosis [83]. Interestingly, Chen *et al*demonstrated that SIRT3 activation with RSV in vitro and in vivo ameliorated collagen deposition, preventing cardiac fibrosis and improving cardiac function in mice.

Overall, these observations suggest that mitochondrial dysfunction is likely to play a key role in the development of fibrotic conditions and can accelerate the process of aging. However, the therapeutic potential of targeting pathways such as activation of SIRT3 via the TGF-β/Smad3 pathway offer exciting new avenues to combating cardiac and pulmonary fibrosis.

## 5.Summary

Aging is an important predisposing factor for fibrotic heart and respiratory diseases. Age-related processes such as senescence and inflammaging diminish the regenerative capacity of damaged cardiac and pulmonary tissue, increasing the likelihood of pathological fibrosis following injury or challenge. What is interesting about these two processes is that at low levels, they mediate beneficial effects, but as you age and the level increases, they become deleterious. This is most evident with senescence, which protects the organism from cancer but which, in excess, can promote aging and the hallmark features of fibrosis. Furthermore, inflammaging and its sustained increase of inflammatory markers, which at normal levels regulate the immune response, contributes to the acquired resistance of myofibroblasts to apoptosis, and the low grade chronic inflammation which sustains the persistent fibrosis of CVD and IPF.

Recycling through autophagy, may offer a pharmacological target in age-related lung and heart fibrotic diseases. However, as described above further investigations are required to fully understand the mechanisms of chronic fibrosis, importantly the contrasting effects of autophagy dysregulation in the heart and lung. Finally, promotion of mitochondrial turnover and a prevention of the imbalances to fission and fusion that trigger that mitochondrial dysfunction may play a key role in preventing the development of fibrotic conditions and the process of aging as demonstrated with SIRT3 activation and the improved collagen deposition. Given the similarities between cardiac and pulmonary fibrosis, investigating targets and testing future treatments in both organs with a focus on these key age-related processes seems justifiable and may lead to better treatment opportunities for age-related cardiac and pulmonary fibrosis. We conclude that ever more sophisticated approaches, and an ever-expanding understanding of the mechanisms underlying the hallmarks of aging will facilitate future interventions for improving human longevity and preventing the onset of fibrosis.
